# Quantification of photooxidative defects in weathered microplastics using ^13^C multiCP NMR spectroscopy†

**DOI:** 10.1039/d2ra00470d

**Published:** 2022-04-07

**Authors:** Anika Mauel, Björn Pötzschner, Nora Meides, Renée Siegel, Peter Strohriegl, Jürgen Senker

**Affiliations:** Department of Inorganic Chemistry III, University of Bayreuth, Universitätsstraße 30 95447 Bayreuth Germany Juergen.senker@uni-bayreuth.de; Department of Macromolecular Chemistry I, University of Bayreuth, Universitätsstraße 30 95447 Bayreuth Germany Peter.strohriegl@uni-bayreuth.de

## Abstract

Weathering of microplastics made of commodity plastics like polystyrene, polypropylene and polyethylene introduces polar polymer defects as a result of photooxidation and mechanical stress. Thus, hydrophobic microplastic particles gradually become hydrophilic, consisting of polar oligomers with a significant amount of oxygen-bearing functional groups. This turnover continuously changes interactions between microplastics and natural colloidal matter. To be able to develop a better understanding of this complex weathering process, quantification of the corresponding defect proportions is a first and essential step. Using polystyrene, ^13^C enriched at the α position to 23%, we demonstrate that ^13^C cross polarisation (CP) NMR spectroscopy allows for probing the typical alcohol, peroxo, keto and carboxyl defects. Even the discrimination between in- and end-chain ketones, carboxylic acids and esters as well as ketal functions was possible. Combined with multiCP excitation, defect proportions could be determined with excellent accuracy down to 0.1%. For materials with ^13^C in natural abundance, this accounts for a detection limit of roughly 1%. The best trade-off between measurement time and accuracy for the quantification of the defect intensities for multiCP excitation was obtained for CP block lengths shorter than 250 μs and total build-up times longer than 2 ms. Further measurement time reduction is possible by using multiCP excitation to calibrate intensities obtained from series of ^13^C CP MAS NMR spectra. As photooxidation is an important degradation mechanism for microplastics in the environment, we expect these parameters to be transferable for probing defect proportions of weathered microplastics in general.

## Abstract 2

We demonstrate an efficient strategy to characterise weathering-induced photooxidative defects in microplastics. The central ^13^C cross polarisation NMR spectra offer high resolution and are quantitative when combined with multiple excitation.

## Graphical abstract

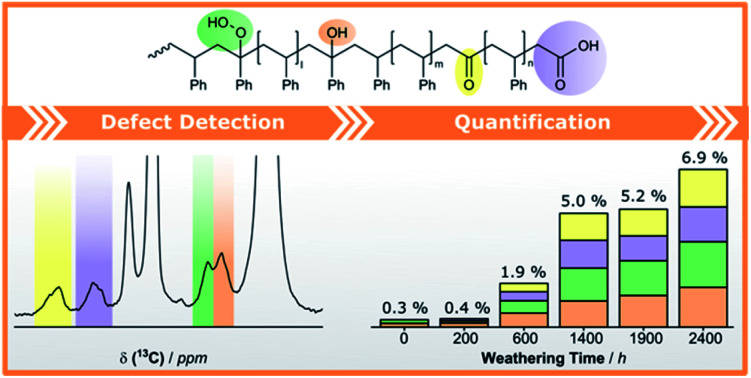

## Introduction

Like for no other material class, polymer materials feature a uniquely broad range of applications due to their low production costs and favourable, easily adjustable properties. Meanwhile, plastic production reaches more than 350 million tons per year,^1^ with more than half of this amount belonging to commodity plastics like polypropylene (PP), polyethylene (PE) and polystyrene (PS) for single-use applications.^1–4^ Insufficient waste management causes large quantities of plastic materials to be released into the environment.^5^ Once in the environment, plastic is expected to persist for ages due to its inherently high durability and chemical inertness. It accumulates and is intensely discussed to pose substantial risks for whole ecosystems.^6–9^ In particular, small particles with dimensions in the micrometre regime are likely to be ingested by sea- and wildlife, inevitably entering the global food chain.^10,11^ Such particles were defined as microplastic (MP), if their dimensions are smaller than 1 mm.^12^ MP particles were found in substantial amounts in aquatic and terrestrial environmental compartments.^13,14^

Degradation of plastics in natural environments plays an important role in understanding the interaction of MP particles with their abiotic and biotic surroundings.^12,15,16^ Starting from hydrophobic materials, the combination of fragmentation and photooxidation creates substantial numbers of chain scissions, polar defects and crosslinks.^17–19^ Weathering gradually changes MP polymer chains to highly polar, heavily branched, medium-sized oligomers.^17^ Polar and charged groups expressed on particle surfaces favour particle cell interactions and cellular responses,^20^ as well as the formation of an ecocorona by adsorption of natural inorganic and organic colloidal particles^21,22^ or by deposition of biofilms due to microbe activity.^23,24^ This in turn enhances the uptake by microorganisms.^25,26^ Considering that, *e.g.*, weathered PS MP particles accumulate hazardous peroxo groups with proportions of several per cent,^17^ the uptake of weathered MP is likely to have negative effects on the organisms.^26,27^ Additionally, the small particle sizes enable fast transport and thus a global distribution.^22,28–30^

The underlying degradation mechanisms of commodity plastics are meanwhile well understood.^2,3,17–19,31–33^ PE, PP and PS consist of an aliphatic backbone and aliphatic or aromatic side chains. Light- and stress-induced radical formation in the presence of oxygen leads to (photo)oxidation, subsequent chain scissions and crosslinking (Fig. S4†).^18,34–36^ As a consequence, a broad variety of functional groups is formed, with the most frequent ones being alcohols, peroxo units, aldehydes, keto groups, carboxy functions and vinyl units.^17,18,37–41^ The individual defect proportions depend on several factors like chemical composition, solar and stress intensity, oxygen fugacity, salinity, temperature fluctuation and more. As the defect types vary in polarity and the ability to form charges as a function of the pH, a prediction of MP physicochemical properties and subsequent model building is not possible without quantifying the defect proportions.^17^

Typical defect proportions are on the order of a few per cent, which makes their quantification challenging.^17,18^ In the past, mostly Fourier Transform Infrared (FTIR), Raman (Ra) and Nuclear Magnetic Resonance (NMR) spectroscopy were used for this purpose.^3,17,20,36,42,43^ FTIR and Raman spectroscopy are very sensitive and are able to probe defect proportions down to a few per mill, if optimal conditions are reached.^3,41^ Nevertheless, the detection limit for Ra spectroscopy is influenced by fluorescence.^41^ FTIR spectroscopy is often used in a semiquantitative fashion by reporting a carbonyl index, which estimates the integral proportion of carbonyl-containing functional groups. Several methods are established to determine the carbonyl index, each leading to a range of possible values.^44^ Especially, when the attenuated total reflection (ATR) technique is used, the results become surface dependent and require concentration-dependent calibration.^3,41^*E.g.*, for weathered LDPE particles, significantly higher carboxyl proportions were detected with FTIR spectroscopy compared to ^13^C MAS NMR spectroscopy, which was attributed to a preferential accumulation of carboxyl groups close to the surfaces.^45^ NMR spectroscopy is an element sensitive bulk method and can be used in a quantitative fashion.^46^ If the samples are soluble, ^1^H liquid-state NMR spectroscopy provides sufficient sensitivity.^42^ Nevertheless, an unambiguous assignment is challenging, as most photooxidation defects consist of quaternary carbon atoms.^20,47^ Using ^13^C as probe instead, provides a better resolution, however, reduces the sensitivity and often requires labelling. Additionally, signal intensities are influenced by cross relaxation, as the decoupling times, during which the proton bath is saturated, are on the order of the spin–lattice relaxation times.^34,37,40,48^ They are also influenced by anisotropic spin–spin relaxation, due to the medium slow polymer dynamics.

Polymer defects were also probed using high-resolution ^13^C solid-state NMR spectroscopy.^38,39,49–51^ As cross relaxation between ^1^H and ^13^C is inefficient for the typically short free induction decay (FID) times, single-pulse (SP) excitation promises sufficient resolution and quantitative intensities.^38,39,50^ Slow spin–lattice relaxation, however, leads to extremely long measurement times. Most studies thus rely on ^13^C labelling for probing the weak defect signals. Alternatively, ^1^H–^13^C cross polarisation (CP) was applied to enhance sensitivity and reduce measurement times.^48,49^ As CP excitation is not quantitative, this approach requires calibration.^48,49^ As an alternative to the insensitive but quantitative SP excitation, recently, the multiCP technique was successfully invoked.^17^ MultiCP relies on a series of short, successive CP bursts separated by a short waiting period, where the polarisation is stored.^52^ In this way, *T*_1*ρ*_ effects are minimized, allowing the signal intensity of carbon species with small build-up rates to develop properly. ^1^H–^13^C multiCP NMR spectra are, therefore, expected to obtain a quantitative character, if the total polarization transfer times are long enough, and were used for a broad variety of materials covering porous, organic and hybrid materials as well as polymers.^17,53–58^ In particular, for chemical units with small effective heteronuclear dipole sums caused by low proton densities in their vicinity^17,53,56,57^ and fast molecular dynamics,^52,54^ significant improvements with deviations from the quantitative intensities below 10% could be reached.^57^

The quantitative determination of proportions, as low as the ones observed for photooxidative defects, is even more challenging with ^13^C MAS NMR spectroscopy. Although recently, multiCP excitation was applied to determine quantitative ^13^C MAS NMR spectra of weathered PS, the low sensitivity coming along with natural abundance prevented to optimise the experimental conditions for the defect resonances themselves.^17^ However, previous studies suggest that this is crucial.^51–57^ Depending on the material properties, the number of CP blocks and the block length needed to be varied between 3 to 10 and 15 μs to 1 ms. Therefore, we performed a case study, evaluating the performance of multiCP for the defect quantification of weathered PS MP particles. These were prepared from partially ^13^C enriched PS (PS-^13^C) by accelerated weathering. The obtained results provide guidelines for the optimal setup of multiCP experiments in order to excite the markedly different defects introduced upon weathering. As the defect types are similar for most commodity plastics like PE and PP, our results impact on the quantification of polymer defects for a major part of weathered MP in the environment.

## Experimental

### Polystyrene: synthesis

PS-^13^C was synthesised by radical polymerisation (Fig. S1†) of 80% styrene (Sigma Aldrich, Missouri, United States of America) and 20% styrene-α-^13^C (99% ^13^C enriched in α position, Sigma Aldrich). While the stabiliser 4-*tert*-butylcatachol was removed *via* an Alox column from styrene, styrene-α-^13^C was used without further purification, thus containing small amounts of 4-*tert*-butylcatechol as stabiliser. The radical initiator azobisisobutyronitrile (AIBN) was freshly recrystallized prior to use. 4.4 ml styrene (38 mmol, 0.80 eq.), 1.1 ml styrene-α-^13^C (9.5 mmol, 0.20 eq.) and 68 mg AIBN (0.48 mmol, 0.010 eq.) were mixed in a Schlenk flask under dry argon (Ar 5.0, Riessner-Gase, Germany) atmosphere and were degassed applying three freeze–pump–thaw cycles. The reaction mixture was heated to 60 °C and stirred overnight for 20 h. The solid product was dissolved in 45 ml toluene at 80 °C. Subsequently, the dissolved polymer was precipitated in 1 l methanol, filtered off and dried in a drying chamber at 40 °C under vacuum. 4.5 g (91% yield) PS-^13^C was obtained with an enrichment degree of 23% at the α-carbon as determined by quantitative ^13^C MAS NMR spectroscopic experiments. It was ground using an Ultra Centrifugal Mill ZM 200 (Retsch GmbH, Haan, Germany) and sieved with an Alpine Air Jet Sieve E200 LS (Hosokawa Alpine AG, Augsburg, Germany) resulting in 125–200 μm sized particles.

### Accelerated weathering

The accelerated weathering experiments were carried out under controlled laboratory conditions in a test chamber Q-SUN XE-3 (Q-LAB Corporation, Westlake, OH, USA) according to a protocol developed previously.^17^ The weathering chamber is equipped with three xenon arc lamps and a Daylight-Q filter system. The latter filters radiation with wavelengths smaller than 295 nm. The emitted radiation is thus similar to natural sunlight (comparison of the emission spectrum to sunlight is given in Fig. S2†). The irradiance was set to 60 W m^−2^ at 300–400 nm, corresponding to 594 W m^−2^ total irradiance (for calculation of the irradiance refer to Table S2†), and the relative humidity was adjusted to 50%. The chamber-irradiance is five-fold enhanced in comparison to the average mid-European irradiation of 114 W m^−2^ (Fig. S3 and Table S2†). 2 g of PS-^13^C particles were weighed into a quartz glass beaker and covered with a quartz-glass lid to enable light penetration from all sides. The particles were constantly stirred in 200 ml deionized water (55 °C) using a PTFE-coated magnetic stirrer at 150 rpm. Particle samples with a weight of 0.3 g each were taken for analysis after 0 h, 200 h, 600 h, 1400 h, 1900 h, 2400 h from the MP-water-dispersion, filtered with a suction filter, and dried at 40 °C in a vacuum oven.

### Solid-state NMR spectroscopy

^13^C MAS NMR spectra were acquired with a Bruker Avance-III HD spectrometer operating at a B_0_ field of 9.4 T corresponding to Larmor frequencies *ν*_0_ of 400.01 MHz and 100.62 MHz for ^1^H and ^13^C, respectively. Samples were spun in a commercial 3.2 mm triple resonance probe. The ^13^C NMR spectra were referenced with respect to tetramethylsilane (TMS) using the secondary standard adamantane. Cross polarisation (CP) experiments were carried out with a ramped ^1^H–^13^C CP sequence at a spinning frequency of 20 kHz, a recycle delay of 3 s and a contact time of 2.0 ms, except for the contact time dependent measurements, where the contact time was varied from 100 μs to 15 ms. The corresponding nutation frequency *υ*_nut_ for the ^13^C channel was set to 50 kHz. The power for the shaped pulse (linear ramp from 50% to 100%) on the ^1^H channel was adjusted to maximal polarisation transfer. ^1^H–^13^C multi-pulse CP experiments were recorded with a sequence (Fig. S5†) published in ref. 51 for PS-^13^C weathered for 1900 h and 2400 h, respectively. While contact times and number of blocks were varied, the Hartman–Hahn conditions and the recycle delay were adopted from the ^1^H–^13^C CP NMR experiments and for *t*_*z*_ 2 s and 3 s were used, respectively. A list of the performed experiments is given in Table 1. The (90°)_−_*_x_* pulses at the end and the beginning of each CP block were set to 2.5 μs (^1^H) and 3.8 μs (^13^C). During acquisition, proton broadband decoupling was applied with a spinal-64 sequence (*υ*_nut_ (^1^H) = 70 kHz). The spectra were deconvoluted using pseudo-Voigt profiles within the simulation package SOLA included in TopSpin 3.2 (Bruker).

**Table 1:** Conducted multiCP ^13^C MAS NMR spectroscopic experiments for pristine and two weathered samples of PS-^13^C

Weathering time	Number of blocks, *n*	Block length, *τ*^block^_multiCP_	Total pol. time, *τ*^tot^_CP_	Waiting time, *t*_*z*_
0 h	12	250 μs	3000 μs	3 s
2400 h	20	150 μs	3000 μs	2 s
	13	150 μs	1950 μs	2 s
	13	150 μs	1950 μs	3 s
	20	150 μs	3000 μs	3 s
	12	250 μs	3000 μs	3 s
	6	500 μs	3000 μs	3 s
	3	1000 μs	3000 μs	3 s
	8	250 μs	2000 μs	3 s
1900 h	8	250 μs	2000 μs	3 s

## Results and discussion

Recently, we proposed a strategy for the quantification of defect proportions based on ^13^C multiCP NMR spectroscopy.^17^ However, due to the extremely long measurement times of several days for one sample, a thorough quality assessment of the applied strategy could not be carried out, in particular not for the weak signals of the polymer defects themselves. By preparing the model system PS-^13^C (partially enriched with ^13^C at the α carbon) we could reduce the measurement time by a factor of ≈400 corresponding to the enhancement of NMR active ^13^C nuclei. Since the majority of photooxidative defects are introduced at the α position (Fig. S4†),^17,18,37,38,47^ we achieve a markedly higher sensitivity for probing the defects. This allows for a systematic exploration of the parameter space of the multiCP experiment, in order to determine the accuracy and reliability of the derived defect proportions.

### Synthesis and qualitative analysis of weathered PS-^13^C

**Fig. 1:**
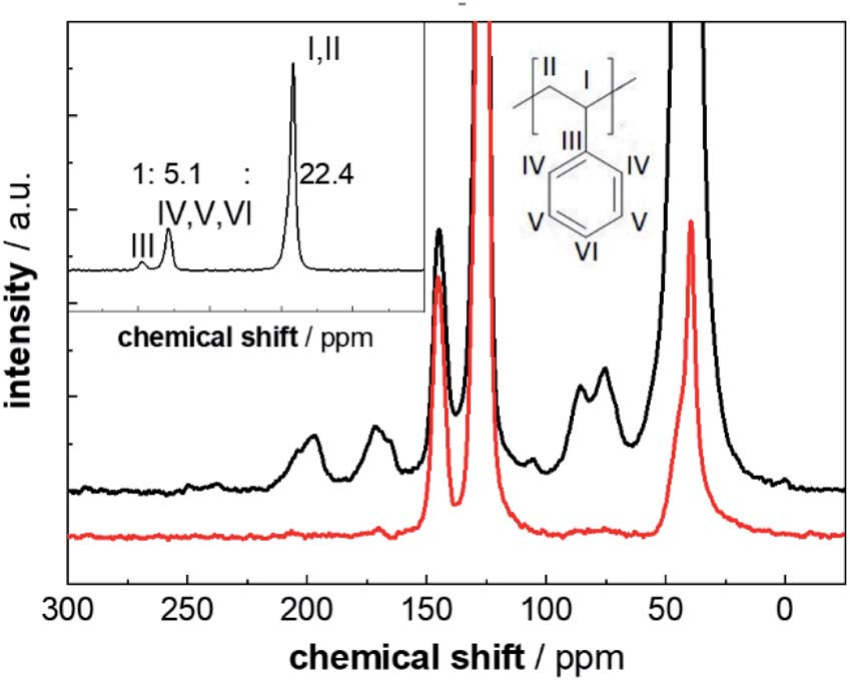
Comparison of ^13^C CP MAS NMR spectra of PS-^13^C (black) and PS in natural abundance (red) of the particle samples weathered for 2400 h. Both spectra are normalized to the intensity of the ipso carbon resonance at 149 ppm and were shifted horizontally for a better overview (full-scale spectra are given in Fig. S6†). Inset: ^13^C multiCP MAS spectrum of pristine PS-^13^C. The relative intensities for the resonances of the ipso carbon(iii), the aromatic carbons (IV, V and VI) and the aliphatic carbons (I (enriched), II) were determined by deconvolution.

**Fig. 2:**
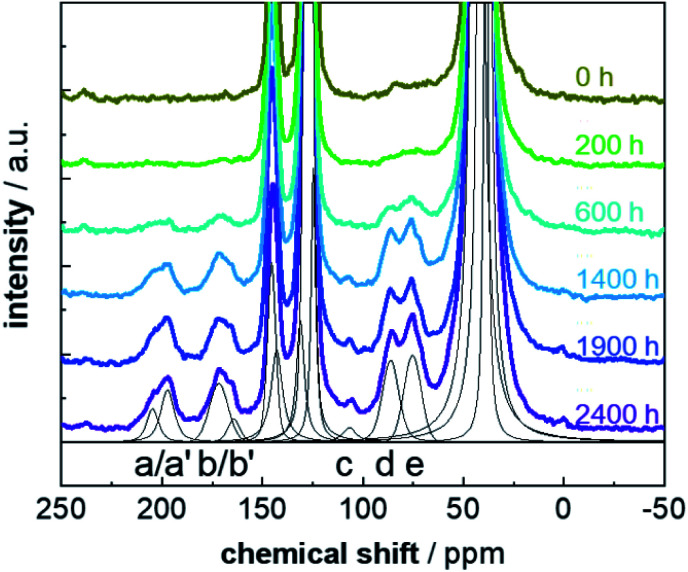
^13^C CP MAS NMR spectra of PS-^13^C for weathering times between 0 h and 2400 h. The functional groups are marked; (a) in-chain and (a') end-chain ketones, (b) carboxylic acids and (b') esters, (c) ketals, (d) peroxides and (e) alcohols. The spectra for the individual weathering times are normalized and horizontally shifted for a better overview. An exemplary fit of the different peaks of the spectra with pseudo-Voigt functions is shown in black for a weathering time of 2400 h.

PS-^13^C was synthesized by radical polymerization using a mix of styrene-α-^13^C (99% enriched), non-enriched styrene, in a proportion of one to four, and a radical initiator. The resulting PS-^13^C exhibits a number average molecular weight *M*_n_ of 51 500 g mol^−1^, a weight average molecular weight *M*_w_ of 194 500 g mol^−1^ and a dispersity *D* of 3.78. A quantitative ^13^C multiCP NMR spectrum (Fig. 1 inset) of the pristine material shows the typical peaks for the ipso carbon at 149 ppm, the aromatic CH groups at 130 ppm and the aliphatic backbone consisting of CH_2_ and CH units at about 43 ppm with intensity ratios of 1 : 5.1 : 22.4. The resonances of the aliphatic groups are strongly superimposed due to the high signal intensity of the partially ^13^C enriched CH groups. Similar intensity ratios were obtained for ^13^C multiCP NMR spectra of different weathering times (Fig. 2 and Table S5†). This ratio corresponds to approximately 23% ^13^C enrichment for the aliphatic CH carbons (ESI chapter S2†). PS-^13^C particles were weathered following the same protocol as previously used.^17^Fig. 1 shows the intensity improvement for the defect signals due to ^13^C labelling compared to the non-enriched sample. Both samples were weathered for 2400 h and the CP spectra were adjusted to one day measurement time and are normalised to the intensity of the ipso carbon. Within the obtained accuracy for weathered PS in natural abundance the intensity gain for PS-^13^C matches the expectation due to the isotope enrichment, demonstrating that the defects are indeed introduced mostly at the α position (Fig. S4†). For dark control experiments (same experimental setup, but without irradiation) carried out on commercial PS with ^13^C in natural abundance, no degradation takes place, which is demonstrated by the unchanged particle sizes and molecular weight distributions as function of the exposure time.^17^ This proves that irradiation is crucial to introduce oxidation defects for the chosen controlled weathering conditions.

^13^C CP MAS NMR spectra acquired for a set of PS-^13^C samples after various weathering times are shown in Fig. 2. The improved S/N ratios allow for a more detailed signal assignment due to a better resolution and the possibility to apply spectral editing by contact-time dependent CP measurements. Even for short weathering times, the seven typical resonances (a–e) of the emerging defects were observed. Surprisingly, also for the pristine PS-^13^C resonances characteristic for alcohol and peroxide units (region around 70–90 ppm) were found. This suggests that already the processing, *e.g.* the grinding, of the samples introduces some defects, due to the emergence of radicals in the presence of strong mechanical forces,^59^ as PS-^13^C is free of any additives.

**Fig. 3:**
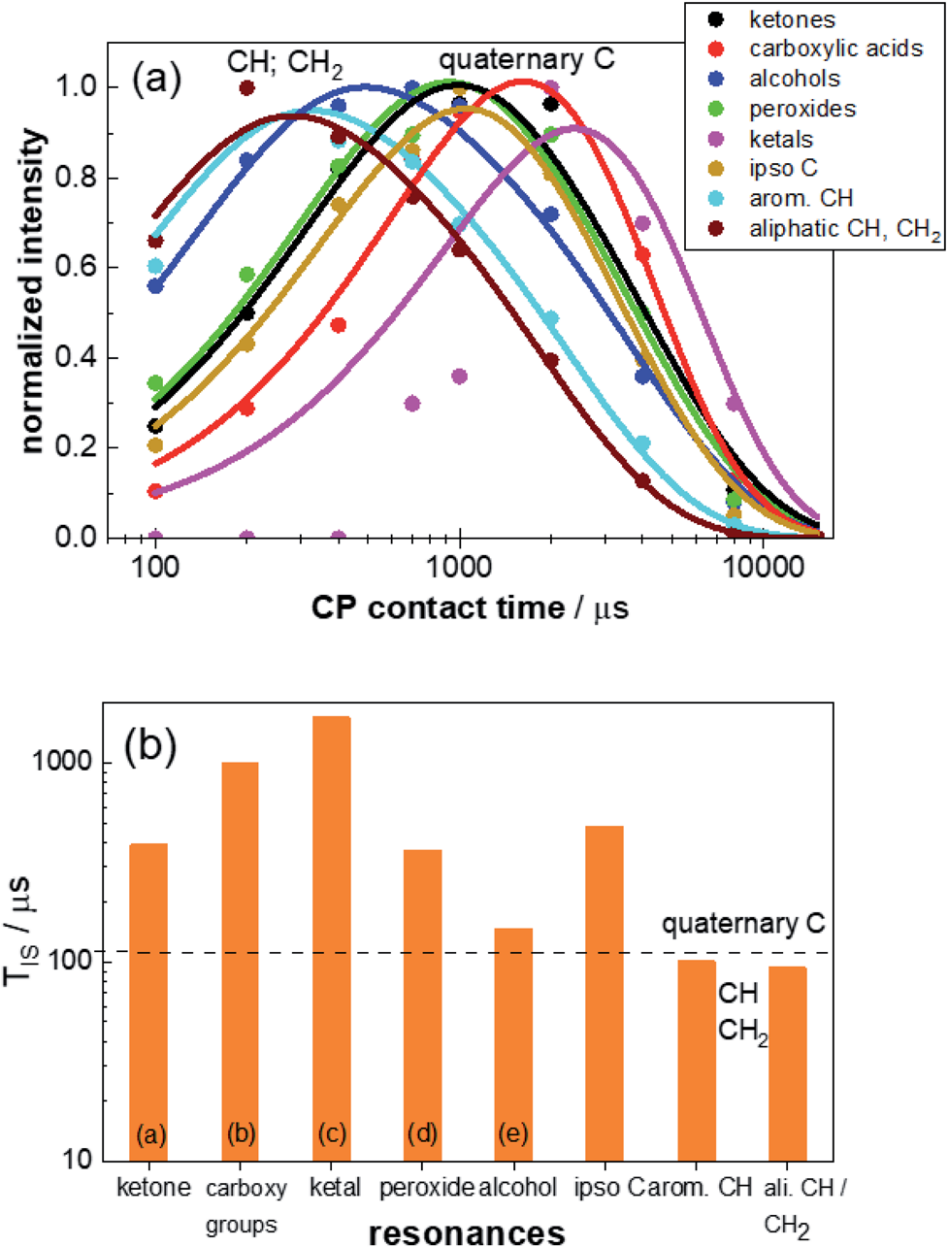
(a) Contact-time dependent intensities of the characteristic resonances for weathered PS-^13^C. The intensities were derived by deconvolution of the ^13^C CP NMR spectra acquired with contact times between 100 μs and 15 ms. The build-up curve for each defect signal (circles) was normalized to the maximum intensity and refined using eqn (1) (solid lines). The determined values for *T*_IS_ and *T*^1H^_1*ρ*_ are given in Table S3.† (b) CP build-up time *T*_IS_ derived from the contact-time dependent build-up curves obtained from the refinements of the CP spectra for the different resonances: (from left to right: ketone groups (a), carboxy groups and esters (b), ketals (c), peroxide groups (d), alcohols (e), ipso carbon, aromatic CH carbons, aliphatic CH and CH_2_ carbons). The shoulders in resonance (a) and (b) were evaluated together with the main peaks of resonance (a) and (b), respectively, since for short CP build-up times shoulder and main peak could not be resolved and the shape of the resonances for longer build-up times does not change for different build up times (Fig. S7†). Due to the heavily superimposed resonances for the aliphatic CH and CH_2_ groups, these two signals were also treated with one build-up curve. Typical *T*_IS_ ranges for quaternary carbons and CH_*n*_ groups are marked by dashed lines.

Contact-time dependent measurements (Fig. 3a) reveal pronounced differences for the build-up behaviour. We use a classic I–S model for the CP build-up^60^ for the refinement. It reads as1
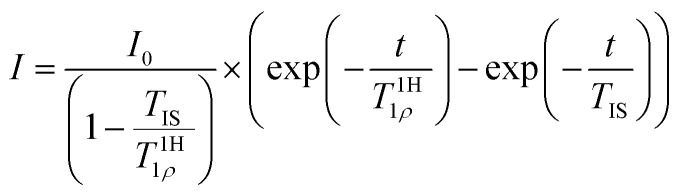
with *T*_IS_ being the CP build-up time, *T*^1H^_1*ρ*_ representing the spin–lattice relaxation time within the rotating frame of the ^1^H bath, and *I*_0_ being the maximum intensity. This equation holds in the limit of abundant protons (I) and *T*_IS_/*T*^13C^_1*ρ*_ ≈ 0, (*T*^13C^_1*ρ*_ being the spin–lattice relaxation time in the rotating frame of the non-abundant (S) carbons), which is usually fulfilled for polymers.^49,59^ We expect *T*_1*ρ*_ in the rotating frame to be proportional to the corresponding *T*_1_ values in the laboratory frame. With *T*_1_(^1^H) = 1.55 s and *T*_1_(^13^C) ≥ 60 s the corresponding *T*^13C^_1*ρ*_ values would be on the order of 100 ms (*T*^13C^_1*ρ*_ ≥ 60 s/1.55 s × *T*^1H^_1*ρ*_) based on the *T*^1H^_1*ρ*_ values obtained from the refinement (Table S3†).

The build-up time constant *T*_IS_ varies between 90 μs and 1700 μs (Fig. 3b and Table S3†) and the relaxation time constants of ^1^H within the rotating frame *T*^1H^_1*ρ*_ differ between 1800 μs and 3900 μs (Table S3†). The *T*_IS_ values for the joint refinement of the CH_2_ and CH units of the aliphatic backbone and aromatic CH‘s are smaller than 100 μs and thus within the expected range.^59^*T*_IS_ decreases with the number of protons in the vicinity of a carbon atom, and is particularly small if the protons are covalently attached to carbon atoms. The other eight resonances have longer or much longer built-up times ranging from 150 μs for the alcohol resonance to 1700 μs for a resonance at 110 ppm. This resonance could not be detected in previous studies carried out on samples without ^13^C enrichment due to the small proportion of the corresponding defect. The *T*_IS_ values for these signals agree with the previous assignment to quaternary carbon atoms, not directly bound to a proton.^17,18,37,38^

In this way, we assigned (a) ketones (≈200 ppm), (b) carboxy units (172 ppm), (c) ketals (105 ppm), (d) peroxide groups (85 ppm) and (e) alcohols (76 ppm). Due to the improved S/N ratio, a distinction between the alcohol and the peroxide signals is now possible, proving that both defect types are present in weathered PS, which was an open debate up to now.^17,18,47^ Additionally, the signals for ketone and carboxy groups split into two resonances each (Fig. 2, peaks a/a' and b/b' and Fig S7†). For the ketone signal group, the chemical shift difference of 7 ppm is characteristic for the difference between (a) in- and (a') end-chain ketones. Both types of defects occur, with the low field-shifted in-chain ketones (204 ppm) being less frequent than the end-chain ketones (197 ppm). The intensity ratio of both resonances (1.7 ± 0.3) suggests that roughly 40% in-chain ketones are formed. The splitting for the carboxy resonances implies that, although the majority of –CO_2_R units consist of carboxylic acids (b), also esters (b') are formed. Their intensity ratio of 4 ± 1 shows that approximately 20% ester groups are formed. The resonance at 105 ppm (c) expresses the typical shift range for vinylidene carbon atoms, which might be a result of the disproportionation reaction (Fig. S4†).^18,37^ However, the long *T*_IS_ value of 1.7 ms, contradicts this assignment since it is characteristic for quaternary carbon units. Thus, it is more likely, that this signal is caused by (hemi)ketal formation (Fig. S4†) by a subsequent reaction of the ketones.^61^ This also explains why this resonance occurs only in later weathering stages after formation of a substantial amount of ketones.

The occurrence of ester and ketal functionalities, suggests two additional crosslinking mechanisms. While C–C bond formation by pairing two radicals dominates early weathering stages, the formation of esters and ketals will become important for later stages, as they require the presence of carboxylic acids, ketones and alcohols in substantial amounts. This explains the surprisingly high crosslinking rates previously observed for weathered PS.^17^

### Quantitative analysis

To be able to derive a better understanding of weathered plastic particles, a reasonably fast and accurate quantitative characterisation of photooxidative defects is essential. We recently proposed a strategy for this purpose.^17^^13^C multiCP NMR spectra were recorded for selected, preferably longer weathering times, and ^13^C CP NMR spectra were acquired for the complete set of weathered samples. The quantitative ^13^C multiCP NMR spectra provide a correction factor *c*_*i*_ for each resonance, which is calculated as *c*_*i*_ = *I*^multiCP^_*i*_/*I*^CP^_*i*_ after normalising both the ^13^C CP and multiCP NMR spectra of the calibration sample to the same overall spectral intensity. The intensities of each defect resonance within the ^13^C CP spectra for sample (a) are then corrected by *I*^quant^_*i*_(a) = *c*_*i*_ × *I*^CP^_*i*_(a). The strategy is time efficient, as multiCP spectra take significantly longer to be recorded with similar S/N ratios. For the present study the measurements are four to five times faster. As long as the materials under investigation are comparable, similar filling factors and measurement parameters for all CP experiments are used, this strategy should be applicable to a broad range of polymers and microplastic samples.

The accuracy of the defect proportions thus crucially depends on how accurate the quantitative relative intensities can be determined for the corresponding resonances within the ^13^C multiCP spectra. The high sensitivity provided by PS-^13^C, enables us to explore the parameter space of the multiCP experiment in a systematic fashion, in particular, for the defect signals. Thus, it provides guidelines and trends to improve the accuracy of future experiments on weathered MP. The multiCP NMR sequence (Fig. S5†) used for this work, is adopted from ref. 51. It consists of a series of *n* short CP blocks. Between the blocks, a z-filter stores the ^1^H and ^13^C polarisation along the external magnetic field. It allows decayed ^1^H polarisation to relax according to *T*^1H^_1_ and flips back the ^1^H and ^13^C polarisation into the *xy* plane of the doubly rotating frame before the next CP burst. Thus, already created polarisation does not decay and is added to the one generated within the next block.^51^ If the waiting time *t*_*z*_ during the z-filter is much shorter than the spin–lattice relaxation of ^13^C nuclei and much longer than the one of ^1^H nuclei (*T*^1H^_1_ ≪ *t*_*z*_ ≪ *T*^13C^_1_) and the block length of the individual CP blocks does not become too short, the I–S model can be applied and the intensity build-up of a resonance with a nominal intensity of one follows eqn (2):^54^2
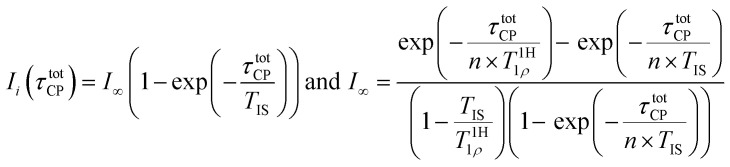


The individual blocks of multiCP experiments should be much shorter than *T*^1H^_1*ρ*_, to avoid relaxation within the rotating frame. With values between 1.8 ms and 3.9 ms obtained for *T*^1H^_1*ρ*_ (Table S3†), we carried out a set of experiments with block lengths from 1 ms down to 150 μs. According to eqn (2), the total contact time *τ*^tot^_CP_ should be on the order of or larger than 3 × *T*_IS_, which accounts for roughly 95% transfer efficiency. With values for *T*_IS_ (Table S3†) between 90 μs (CH_*n*_ groups) and 1000 μs (carboxy units), we chose to record two series of measurements with *τ*^tot^_CP_ either set to 2 ms or 3 ms. Finally, the waiting time *t*_*z*_ during the z-filter should allow for the spin–lattice relaxation of the decayed ^1^H polarisation and should thus be on the order of 2 × *T*^1H^_1_. However, to avoid significant decay of ^13^C polarisation, *t*_*z*_ ≪ *T*^13C^_1_ also has to be fulfilled. *T*^1H^_1_ was determined to 1.5 s and *T*^13C^_1_ was estimated to be larger than 60 s. Therefore, we explored the influence of *t*_*z*_ by collecting ^13^C multiCP NMR spectra with *t*_*z*_ being either 2 s or 3 s. Furthermore, three samples with weathering times of 0 h, 1900 h, and 2400 h were probed. The latter two samples were chosen to guarantee meaningful defect proportions. An overview of all multiCP experiments and values for *I*_∞_ for each resonance (eqn (2)) are given in Tables 1 and S4.† With some limitations, *I*_∞_ might be used to correct the intensities obtained from multiCP experiments. Additionally, it allows to estimate whether the chosen block lengths and number of blocks are appropriate to avoid heavy losses due to *T*^1H^_1*ρ*_. To benefit from eqn (2) though, *T*_IS_ and *T*^1H^_1*ρ*_ need to be determined separately, which might not always be feasible for studies on complex materials like microplastics.

**Fig. 4:**
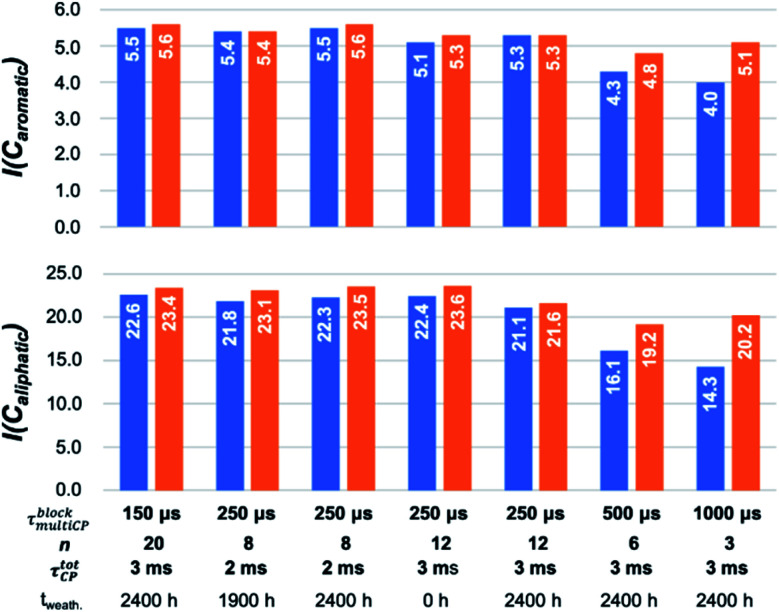
Comparison of the relative intensities for the aromatic CH units (top) and the aliphatic CH/CH_2_ groups (bottom) for various multiCP experiments before (blue) and after correcting with *I*_∞_ (orange). All relative intensities are given after normalising the intensity for the ipso carbon to one (Table S5†). For small block length average relative intensities of 1 : 5.4 : 22.0 for *I*(C_ipso_), *I*(CH_aro_) and *I*(C_aliphatic_) were obtained matching the expected stoichiometry and enrichment degree.

For the chosen block lengths *τ*^block^_multiCP_ and number of blocks *n*, *I*_∞_ varies between 0.60 and 0.98 (Table S4†) suggesting that significant deviations for the intensities are to be expected, in particular for longer *τ*^block^_multiCP_ and smaller *n*. This trend (Fig. 4 and Table S5†) is nicely reproduced for the relative intensities for the three main resonances of PS-^13^C (ipso carbon, aromatic CH and aliphatic CH/CH_2_). For *τ*^block^_multiCP_ of 500 μs and 1 ms (*n* adjusted to yield a *τ*^tot^_CP_ of 3 ms) the relative intensities of the signals of the aromatic CH groups and the aliphatic CH/CH_2_ units are significantly smaller than the expected stoichiometric values (1 : 5 : ≈22). The deviation becomes stronger for longer block lengths. For *τ*^block^_multiCP_ = 1 ms, the aromatic CH and aliphatic units are underestimated by roughly 20% to 30% (Fig. 4).

This demonstrates that the block lengths need to be kept short enough to avoid polarisation loss due to *T*^1H^_1*ρ*_, in particular, for chemical units with small *T*_IS_ (*e.g.* CH and CH_2_) and thus fast build-ups. Consequently, the number of blocks becomes large to adjust the long *τ*^tot^_CP_ necessary to accommodate for quaternary carbon atoms with large *T*_IS_ values (Table S3†). For *τ*^block^_multiCP_ of 250 μs and 150 μs the expected relative intensities were obtained. With respect to a nominal intensity of 1 for *I*(C_ipso_), *I*(CH_aro_) scatters between 5.1 and 5.5 and *I*(C_aliphatic_) lies between 21.1 and 22.6. This implies that convergence is reached and that quantitative intensities and thus proportions can be calculated with an accuracy of roughly 10%, estimated from the variation of the determined relative intensities. As similar relative intensities were also obtained for *τ*^tot^_CP_ = 2 ms, the influence of the total contact-time is less severe in the chosen regime. Especially, for the *τ*^block^_multiCP_ = 150 us measurements (see Fig. S8†), the spectral shape and thus the relative intensities do not change when reducing *τ*^tot^_CP_ from 3 ms (20 blocks) to 2 ms (13 blocks) or when reducing the waiting time *t*_*z*_ to 2 s (Fig. S8†). This indicates that we are already in the limit of sufficient long waiting times *t*_*z*_ and total contact-times *τ*^tot^_CP_.

Using *I*_∞_ to correct for lost polarisation during the individual CP blocks allows for compensating the major part of the polarisation losses (Fig. 4). For ^13^C multiCP experiments with *τ*^block^_multiCP_ = 500/1000 μs average relative intensities of 4.9 for *I*(CH_aromatic_) and 19.7 for *I*(C_aliphatic_) were reached. Although they are close to the plateau values of 5.4 and 22.0 established from the shorter block lengths, the remaining differences are on the order of 10%. They might be caused by deviations from the I–S model due to coherent polarization transfer, which should be strongest for the proton bearing groups. For smaller *τ*^block^_multiCP_, *I*_∞_ approaches unity and thus leads to small corrections only. Within the limit of the aimed for accuracy of 10%, *I*_∞_ might then be neglected. This is important, as for many complex materials the determination of *T*_IS_ and *T*^1H^_1*ρ*_, needed to calculate *I*_∞_, is challenging and would reduce the applicability of multiCP experiments.

**Fig. 5:**
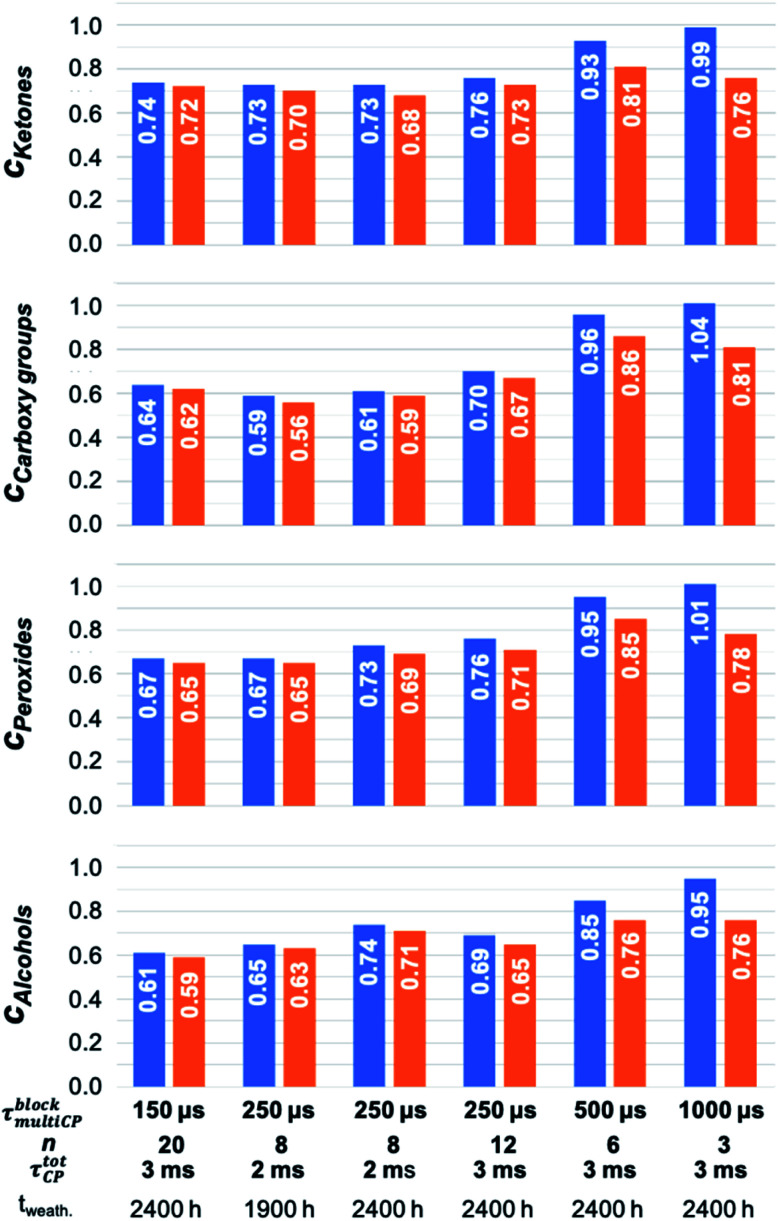
Comparison of calibration factors *c*_*i*_ (blue) for the major polymer defects. The *c*_*i*_ were derived by dividing the intensities of resonance *i*, obtained from the ^13^C multiCP NMR spectrum, by the ones extracted from the ^13^C CP NMR spectrum. Both NMR spectra are normalised to the same total intensity. Calibration factors *c*_*i*_ which are corrected for polarisation loss due to *T*^1H^_1*ρ*_ within the CP blocks according to eqn (2) are given in orange.

**Fig. 6:**
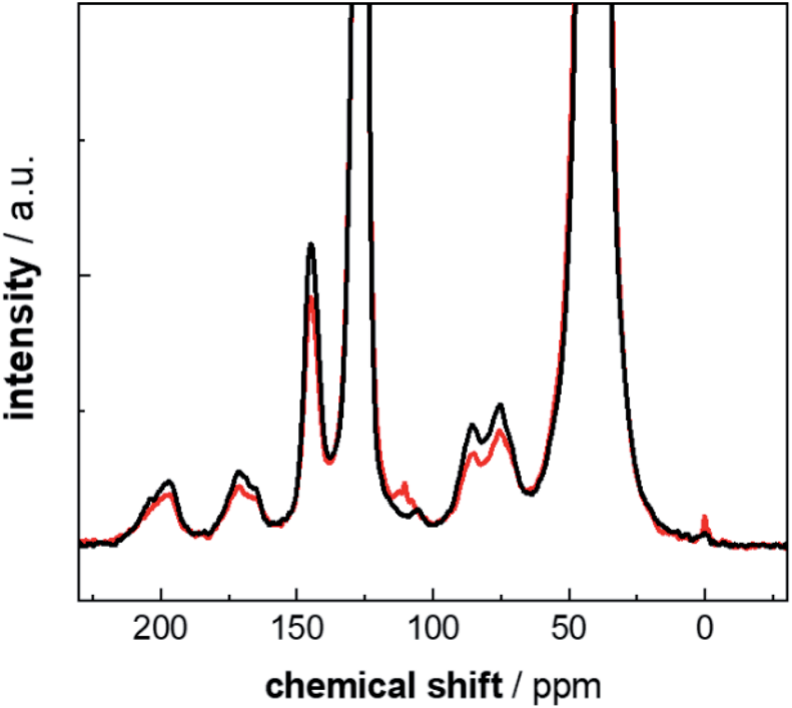
Comparison of ^13^C CP (*τ*^tot^_CP_ = 2 ms; black line) and multiCP ^13^C (*τ*^block^_multiCP_ = 250 μs with 12 blocks and *τ*^tot^_CP_ = 3 ms; red line) MAS NMR spectra for PS-^13^C weathered for 2400 h. Both spectra are normalized to the same total intensity (full scale spectra are given in Fig. S9†).

In a second step, the intensities determined from the ^13^C multiCP NMR spectra were used to calibrate the ^13^C CP NMR spectra. Calibration factors *c*_*i*_ were derived for every resonance as described above (Fig. 5, blue bars and Table S6†). In particular, for the analysis of the defect resonances, the calibration is advantageous, for two reasons. First, a direct comparison to expected proportions is not feasible since the latter are not known in advance. Second, the defect intensities are weak, on the order of roughly 10% of the total accumulated intensity and their intensity changes are easier recognized in the calibration factors *c*_*i*_. Due to its very weak intensity, we exclude the ketal resonance at 110 ppm from this analysis. For comparison, the *c*_*i*_ values were also corrected with *I*_∞_ (Fig. 5, orange bars and Table S7†), to test how well the I–S model (eqn (2)) can be used to account for polarisation losses, if the block length *τ*^block^_multiCP_ of the CP blocks becomes too long. The general CP conditions were chosen to be the same for both the CP and multiCP experiments. To obtain better S/N ratios though, all CP NMR spectra were obtained with a contact time *τ*^tot^_CP_ of 2 ms, due to the markedly higher signal intensity compared to 3 ms. Fig. 6 shows the typical differences between a ^13^C CP (*τ*^tot^_CP_ = 2ms) and a ^13^C multiCP spectrum (*τ*_CP_ = 250 μs with 12 blocks and *τ*^tot^_CP_ = 3 ms). Interestingly, with these conditions the defect intensities derived from the CP NMR spectra are overestimated compared to the quantitative values obtained from the multiCP experiments. As a consequence, all *c*_*i*_ values for the defects are smaller than one (Fig. 5).

As observed for intensities of the main PS-^13^C resonances within the ^13^C multiCP NMR spectra, the *c*_*i*_ values for the defects converge for *τ*^block^_multiCP_ smaller than 250 μs to values between 0.6 and 0.7 depending on the defect type. This trend holds for *τ*^tot^_CP_ of 2 ms and 3 ms as well as for *t*_*z*_ of 2 s and 3 s equally, demonstrating that both total contact-times and waiting times are long enough, to guarantee a sufficient polarisation build-up for the polymer defects. For *τ*^block^_multiCP_ larger than 500, the calibration factors grow and approach one for *τ*^block^_multiCP_ = 1000 μs. Correcting the ^13^C multiCP NMR spectra with *I*_∞_ before calibration improves the *c*_*i*_ values for *τ*^block^_multiCP_ of 500 μs and 1000 μs markedly. For smaller block lengths the correction is negligible. We attribute the increase in *c*_*i*_ for longer *τ*^block^_multiCP_ to polarisation losses for the resonances of the aromatic and aliphatic CH and CH_2_ units. They exhibit the smallest *I*_∞_ of around 0.6 (Table S4†) and thus should suffer the strongest losses.

Underestimating the intensities of the main PS resonances, in turn, leads to an overestimation of the defect intensities and thus an increase for *c*_*i*_. Nevertheless, the corrected *c*_*i*_ values do not reach the plateau derived from the experimental data for the smaller block length, suggesting that additional polarisation losses due to, *e.g.*, *T*^13C^_1*ρ*_ and *T*^13C^_1_ relaxation as well as coherent polarisation transfer, beyond the I–S model, are playing a role. While the calibration factors *c*_*i*_ for the ipso carbon atoms exhibit the same trend as for the defect *c*_*i*_ values, the ones for the aromatic and aliphatic CH and CH_2_ units are apparently independent of the conditions chosen for the multiCP experiments. As they share similar build-up and relaxation behaviour and account for 95% of the total spectral intensity, calibration leads to constant *c*_*i*_ values (Fig. 4).

To show that the optimized experimental conditions for the multiCP experiment (*τ*^block^_multiCP_ = 250 μs, 12 blocks) are meaningful also for samples in natural abundance we remeasured a sample of commercial PS (weathered for 3200 h) from ref. 17, which was previously obtained with a block length of 500 μs and 6 blocks. The comparison between both spectra is given in Fig. S10.† As expected from Fig. 4, the intensity of the aromatic and aliphatic proton bearing groups is enhanced by roughly 20%. Especially the expected intensity ratios 1 : 5 : 2 of the three main polystyrene resonances (ipso C, aromatic CH's and aliphatic CH/CH_2_) are correctly reproduced. In consequence, the total defect proportions amounting to 13% are roughly 20% smaller than the previously reported ones (16.5%).^17^ This matches the expected trends of the correction factors shown in Fig. 5.

**Fig. 7:**
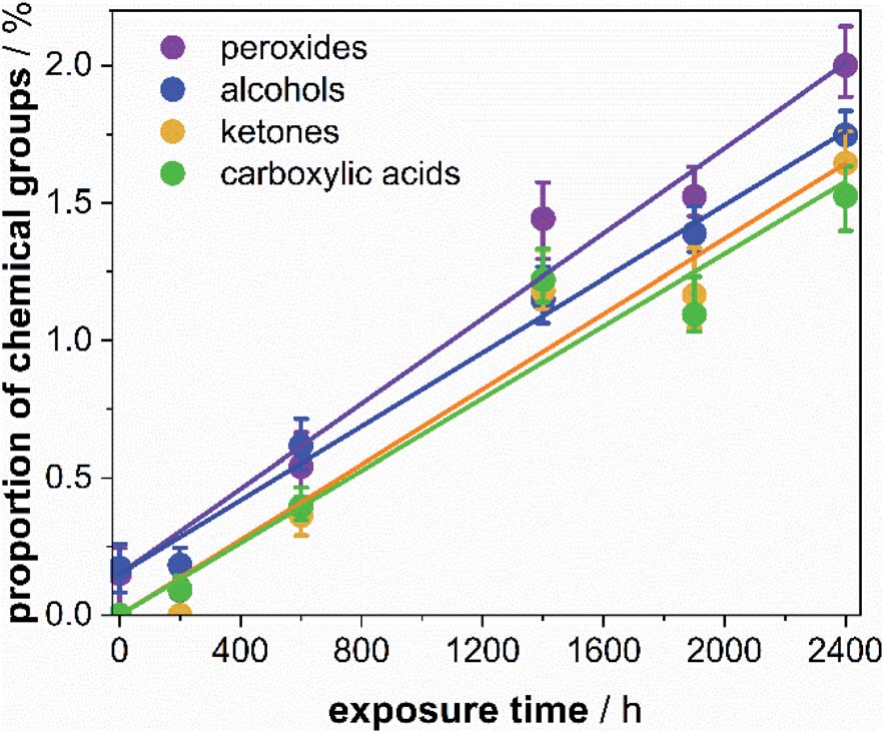
Proportion of the defect groups with respect to the total number of repeating units of PS-^13^C in dependence of the weathering time. The defect proportions were refined with a linear function allowing to derive growth rates for carboxylic acids (6.6 × 10^−6^ h^−1^), ketones (6.9 × 10^−6^ h^−1^), alcohols (6.7 × 10^−6^ h^−1^) and peroxide (7.7 × 10^−6^ h^−1^) functions. The non-zero interception for the peroxides and alcohols is a consequence of the grinding procedure of the pristine PS-^13^C.

Applying the proposed calibration method, the intensities derived from the ^13^C CP MAS NMR spectroscopic experiments were calibrated according to the correction factors for *τ*^block^_multiCP_ = 150 μs and 20 blocks (Table S6†). From these spectral intensities, the proportion of the respective defects was calculated according to Meides *et al.*,^17^ additionally considering the selective ^13^C enrichment of 23% at the α position of PS (see ESI† for calculations). Within the probed weathering time, the proportions for all defect types grow with a linear trend. Rates between 6.6 × 10^−6^ h^−1^ and 7.7 × 10^−6^ h^−1^ were observed, demonstrating that all defect types grow at similar rates. Considering that weathering in our controlled weathering experiment is roughly 5 times faster compared to average central European conditions, the maximum gain of total defect proportions of PS microplastic particles in the environment is extrapolated to roughly 5% per year (Fig. 7). The rates are slightly smaller than observed for commercial non-additivated PS in natural abundance.^17^ For shorter weathering times, the improved S/N ratio allows for probing even very small defect proportions down to 0.1%. In this way, we show that the implied induction period for ketone formation^17^ was caused by a lack of sensitivity. Here, a continuous increase of the ketone proportions was observed.

## Conclusion

For the present study, we synthesised PS-^13^C – polystyrene partially ^13^C labelled at the α position – by radical polymerisation with an enrichment degree of ≈23%. Accelerated weathering by exposure to mechanical stress and simulated solar radiation, then allowed to introduce typical photooxidative defects. They increase in a linear trend as function of the weathering time up to individual proportions of 2%. As most defects originate at the α position, PS-^13^C proved to be a highly sensitive model system for probing defect types and their proportions by combining ^13^C CP and multiCP MAS NMR spectroscopy. While detection *via*^13^C offers the necessary resolution for identifying and distinguishing the defect types, CP excitation promises reasonable measurement times. Calibration with multiCP NMR spectra led to quantitative relative intensities and thus to defect proportions. As the S/N ratio was improved roughly 20 times, we could explore the influence of essential parameters for the multiCP experiment on the intensities of the defects themselves.

In addition to the typical alcohol, peroxo, keto and carboxy functions already described earlier,^17^ the higher sensitivity for PS-^13^C allowed to distinguish between in- and end-chain ketones, carboxylic acids and esters. Even (hemi)ketal functionalities in very low proportions could be identified. The presence of ester and ketal groups suggest that carboxylic acids and ketones react with alcohol units for longer weathering times. They thus introduce two additional crosslinking mechanisms, besides radical pairing and subsequent C–C bond formation. For selected PS-^13^C samples (0 h, 1900 h and 2400 h weathering times), we characterised ^1^H and ^13^C spin–lattice relaxation, which exhibits averaged time constants of 1.5 s (*T*^1H^_1_) and ≈60 s (*T*^13C^_1_) in the laboratory frame and of 3 ms (*T*^1H^_1*ρ*_) and ≈100 ms (*T*^13C^_1*ρ*_) in the double rotating frame. The CP build-up time constants *T*_IS_, determined *via* contact-time dependent measurements, vary markedly from 90 μs for CH/CH_2_ units up to 1 ms for carboxy units. Remarkably, although all defect types are quaternary in nature, already their *T*_IS_ values reach down to 150 μs.

The broad spread for *T*_IS_ requires a careful setup for the multiCP experiments. We explored the influence of essential parameters, like number of CP blocks, their block length and the waiting time between the successive blocks, to determine ideal conditions and guidelines for samples with ^13^C in natural abundance, where an adjustment on the defect resonances themselves is not possible. While the effect on the main resonances of PS-^13^C (C_ipso_, CH_aromatic_ and C_aliphatic_) was best studied on the relative intensities of the ^13^C multiCP NMR spectra, for the defect resonances *i* the calibration factors *c*_*i*_ = *I*^multiCP^_*i*_/*I*^CP^_*i*_ turned out to be more meaningful. We found convergence for all resonances (both main and defects) for total contact-times (*τ*^tot^_CP_) and waiting times (*t*_*z*_) longer than 2 ms and 2 s, respectively. This matches previously reported guidelines,^51,54^ where *t*_*z*_ > 2 × *T*^1H^_1_, *t*_*z*_ < *T*^13C^_1_ and *τ*^tot^_CP_ ≈ 3 × *T*_IS_ should be adjusted. While chemical units with large *T*_IS_ values turned out to be relative insensitive with respect to the CP block length (*τ*^block^_multiCP_), in particular, the intensities for the aromatic and aliphatic CH and CH_2_ units showed large deviations from expected relative intensities for longer block lengths. For a block length of 1 ms already a polarisation loss on the order of 20 to 30% occurred. This effect could be avoided for block length smaller than 250 μs and the best conditions were observed for 150 μs. Thus, to reach a total contact-time larger than 2 ms, a minimum of 8 and 13 blocks is required, respectively.

As weathered MP generally contains a broad mix of defects and functional groups, which may cover the whole range between quaternary and primary carbon atoms, multiCP experiments with block lengths shorter than 250 μs and total contact times longer than 2 ms are advisable for future experiments. This opposes, to a certain extent, previous studies on other complex materials,^51–57^ where often significantly longer block length were used. The necessary short block lengths, however, increase the measurement time markedly, as the number of repetitions will be large. Collecting ^13^C multiCP NMR spectra for whole sample series is thus not feasible. We, therefore, suggest to calibrate conventional ^13^C CP NMR spectra with selectively acquired multiCP experiments. Our results demonstrate that the proposed strategy offers a reasonable fast and accurate way to characterise polymer defects for MP even for ^13^C in natural abundance and for the low inherent defect proportions. We expect that both the calibration and the obtained parameter range are applicable to other types of commodity plastics and thus will help to overcome the challenge of determining average stoichiometries for weathered MP. In addition, the presented approach will be transferable to a broad range of organic, inorganic and hybrid materials supporting structure determination of complex materials.

## Conflicts of interest

There are no conflicts to declare.

## Supplementary Material

RA-012-D2RA00470D-s001
