# Supplementary material for: Quantification of photooxidative defects in weathered microplastics using 13C multiCP NMR spectroscopy
Source: RSC Adv. 2022 Apr 7;12(18):10875–85. doi: 10.1039/d2ra00470d (PMC8988274; doi:10.1039/d2ra00470d)
Supplement: RA-012-D2RA00470D-s001 [file RA-012-D2RA00470D-s001.pdf]

Electronic Supplementary Material (ESI) for RSC Advances.
This journal is © The Royal Society of Chemistry 2022

Supporting Information

for

Quantification of photooxidative defects in weathered microplastics using ^{13}C multiCP NMR spectroscopy

Anika Mauel,[†,a] Björn Pötzschner,[†,a] Nora Meides,[b] Renée Siegel,[a] Peter Strohriegl,[*b] Jürgen Senker[*a]

[a] Department of Inorganic Chemistry III, University of Bayreuth, Universitätsstraße 30, 95447 Bayreuth, Germany

[b] Department of Macromolecular Chemistry I, University of Bayreuth, Universitätsstraße 30, 95447 Bayreuth, Germany

† These authors contributed equally to this work

* Corresponding authors: Peter.strohriegl@uni-bayreuth.de, Juergen.senker@uni-bayreuth.de;

S1 Figures and tables

Synthesis of ^{13}C labelled polystyrene

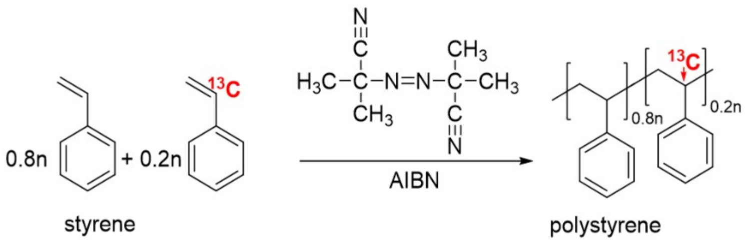

Fig. S1 Synthesis of polystyrene from a 4:1 mixture of ^{13}C labelled and non-labelled styrene with radical initiator AIBN.

Table S1 Educt proportions for PS-^{13}C synthesis sketched in Fig. S1.

**Substance**	**n [mmol]**	**Equivalents**	**m [g]**	**M [g/mol]**	**V [mL]**	**ρ [g/mL]**
**Styrene**	38.04	0.8	3.96	104.15	4.4	0.91
**^{13}C enriched Styrene**	9.51	0.2	1	105.14	1.1	0.92
**AIBN**	0.476	0.01	0.0677	142.25	/	/

Accelerated weathering

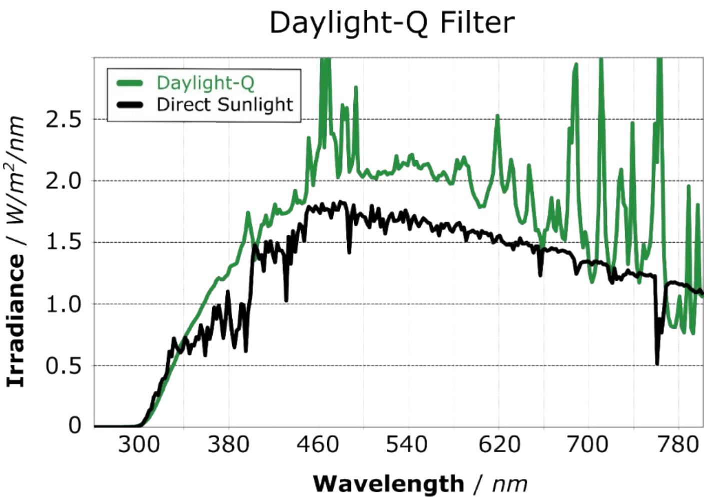

Fig. S2 Radiation spectrum of the weathering chamber created by three Xe lamps and a Q-SUN daylight-Q filter compared to the one of direct sunlight. Taken and modified from Q-Lab Corporation, 2014, Technical Bulletin LX-5060, A Choice of Filters for Q-Sun Xenon Test Chambers.

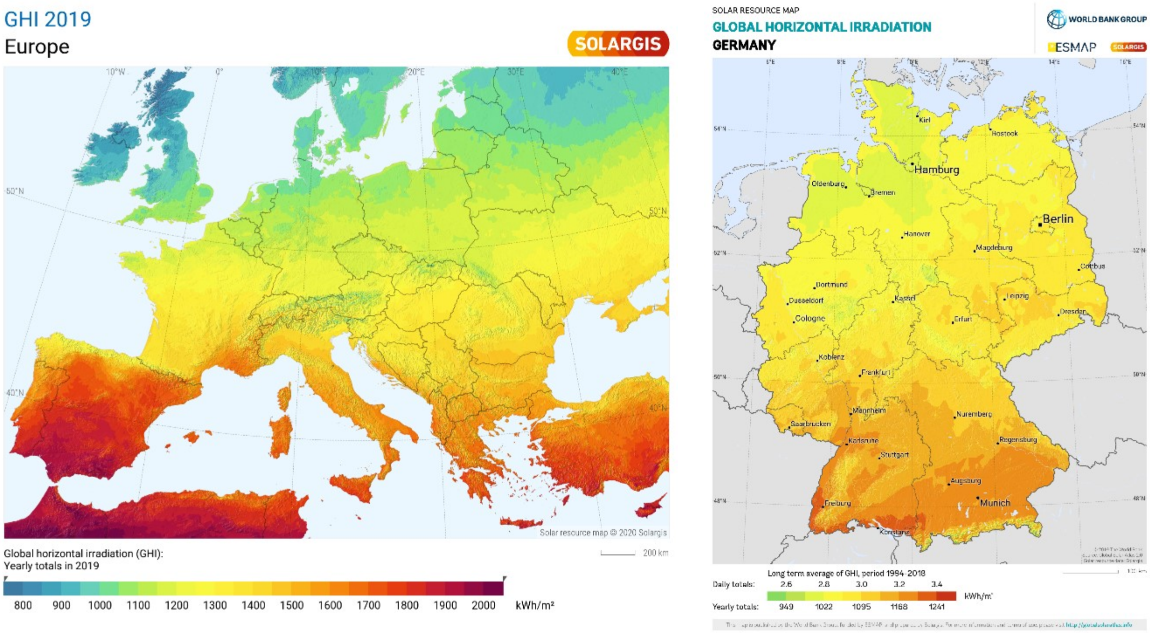

Fig. S3 Global horizontal irradiation for Europe and for Germany in kWh/m2 for 2019. Source: SOLARGIS.

Table S2 Calculation of total irradiance in the Q-SUN Xe-3 weathering chamber as well as the acceleration factor for Central Europe.

**Calculation of total irradiance in Q-SUN Xe-3 chamber**		
Calculation factor*1	9.9	-
Lamp settings	60	W/m^2
Total irradiance in Q-SUN Xe-3 chamber	594	W/m^2
**Acceleration factor of Q-SUN Xe-3 chamber compared to Central Europe**		
Solar irradiance per year*2	1000	kWh/m^2
Hours per year	8765	h
Solar irradiance	114	W/m^2
Acceleration factor of Q-SUN Xe-3 chamber*3	5.21	-

*1 calculation factor extracted from Q-Sun Irradiance Conversion Sheet provided by Q-Lab Germany

*2 value was derived from Figure S3 for the longitude of Central Europe

*3 this value was calculated by division of the total irradiance in the Q-Sun Xe-3 chamber (594 W/m^2) by the solar irradiance determined for Central Europe (114 W/m^2)

Degradation Mechanism

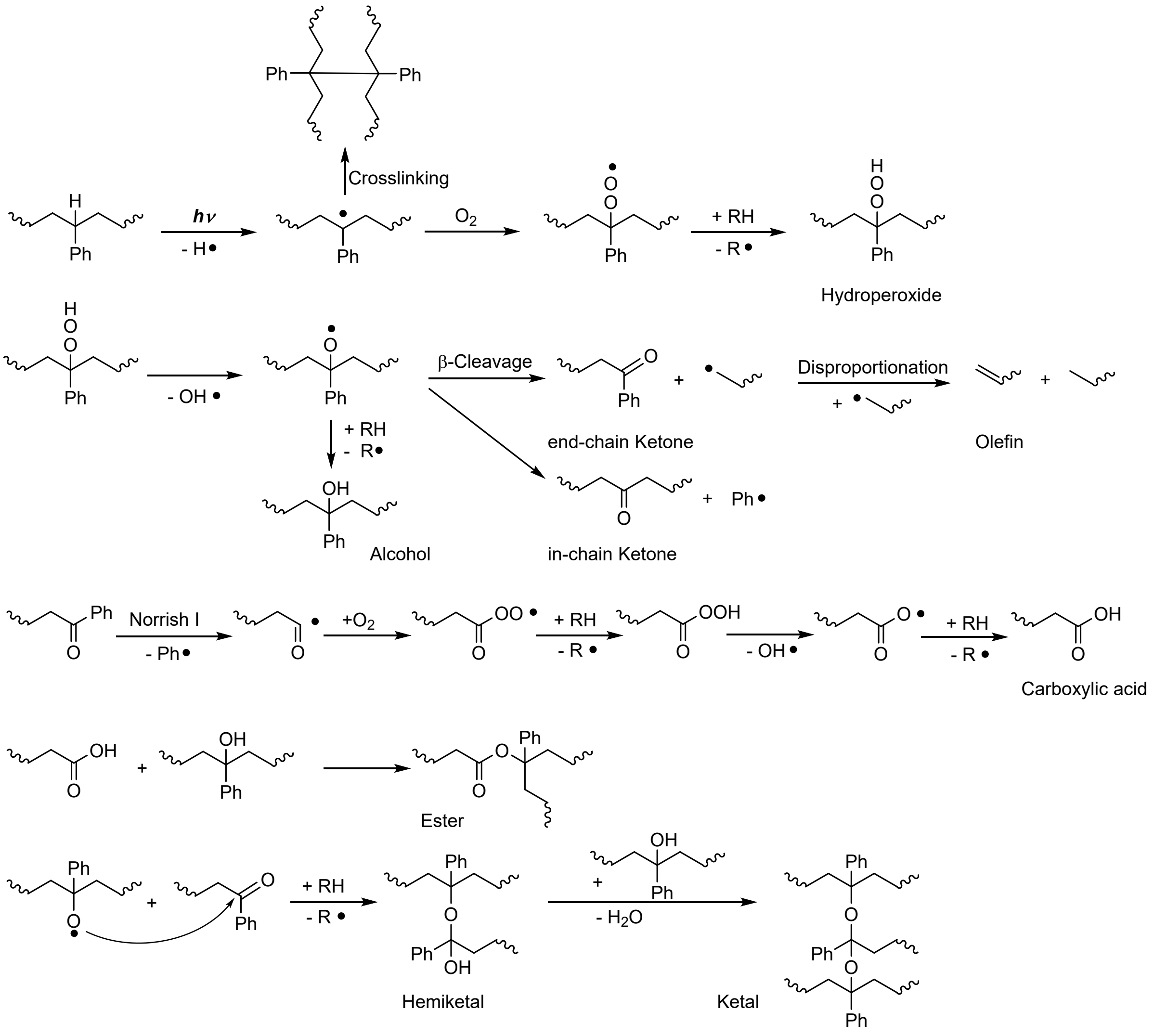

Fig. S4 Typical pathways for photooxidative degradation of PS, outlining the formation of hydroperoxides, alcohols, in-chain and end-chain ketones, carboxylic acids, esters, hemiketals and ketals (see main paper refs.18, 36, 39 and 61).

NMR Spectroscopy

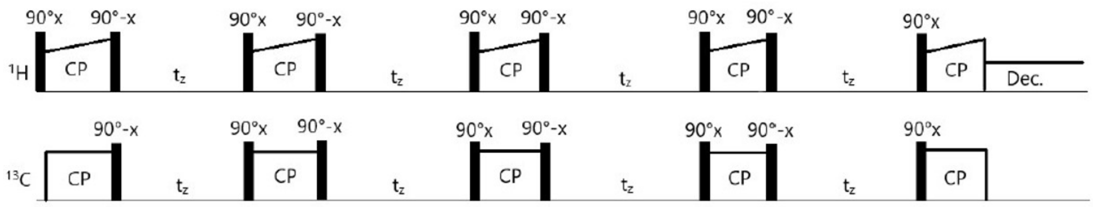

Fig. S5 MultiCP sequence as introduced in ref. 52 used for this work.

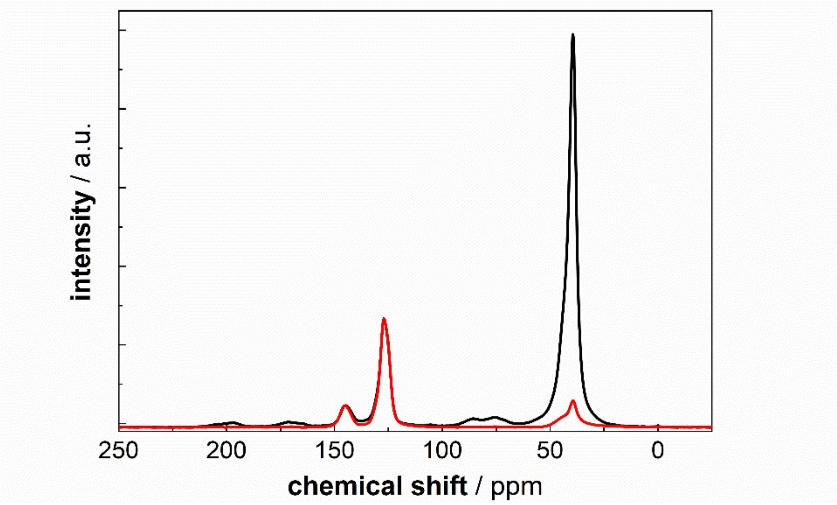

Fig. S6 Comparison of ^{13}C CP MAS NMR spectra of PS-^{13}C (black) and PS in natural abundance (red) of the particle samples weathered for 2400 h. Both spectra are normalised to the intensity of the ipso carbon resonance at 149 ppm.

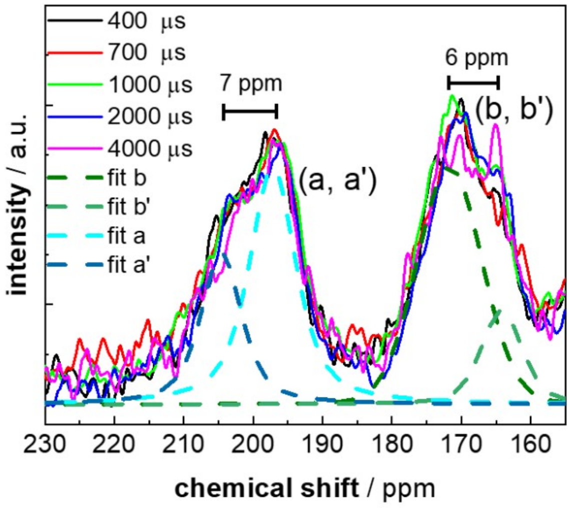

Fig. S7 ^{13}C CP MAS spectra of PS-^{13}C weathered for 2400 h highlighting the spectral range for ketone and carboxy functions for selected CP build-up times (400 µs to 4000 µs). The ketone resonance (a) is split in in-chain (left shoulder, (a), 204 ppm) and end-chain ketones ((a'), 197 ppm). The peak at 172 ppm is attributed to the carboxylic acids (b), the small shoulder at 166 ppm to esters (b'). The relative intensity ratios of the shoulders and the main peaks do not change significantly with the polarization time. The dashed lines represent pseudo-Voigt fits of the individual resonances.

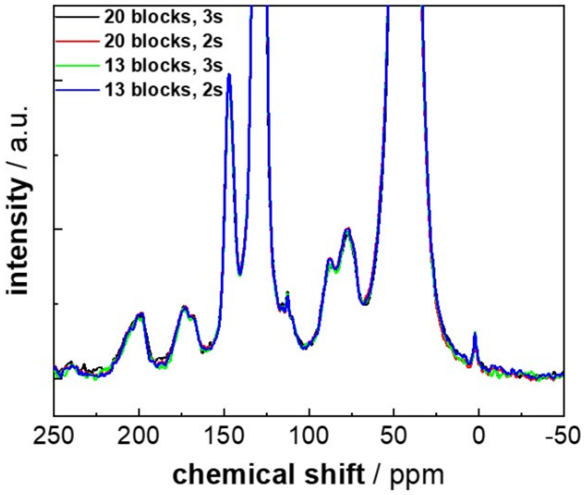

Fig S8 ^{13}C multiCP MAS spectra of PS-^{13}C weathered for 2400 h, measured with a block length of 150 µs, different number of blocks n (13 or 20) and waiting times t_z (2 s or 3 s). The intensities of all spectra are normalized to the intensity of the aliphatic CH/CH_2 resonance. The shape and intensity of the individual resonances are practically independent of the varied measurement parameters.

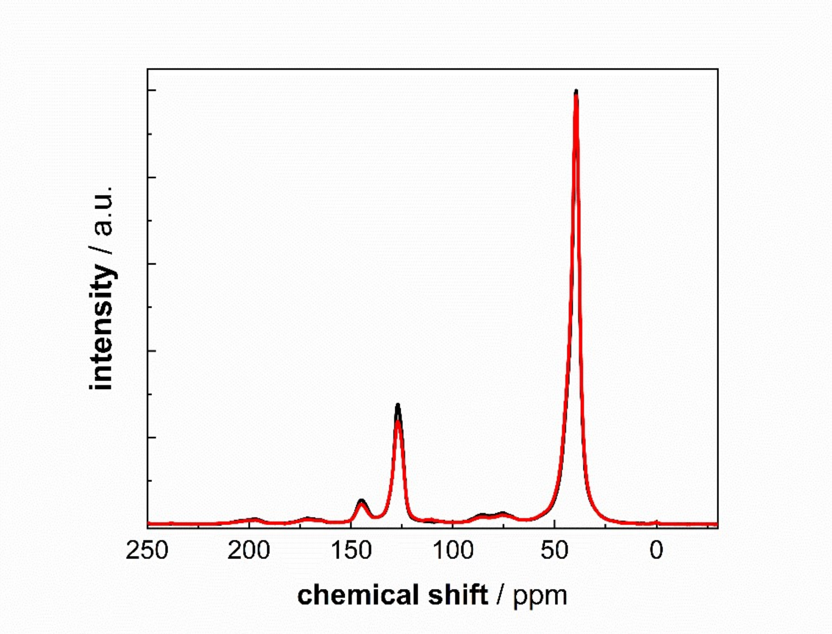

Fig. S9 Comparison of ^{13}C CP (τ_{CP}^{tot} = 2 ms; black line) and multiCP ^{13}C ($\tau_{multiCP}^{block}$ = 250 µs with 12 blocks and τ_{CP}^{tot} = 3 ms; red line) MAS NMR spectra for PS-^{13}C weathered for 2400 h. Both spectra are normalised to the same total intensity. Thus, the individual intensities may vary.

Table S3 Values for T_{IS} and $T_{1\rho}^{1H}$ as determined from the refinements of the build-up curves shown in Fig. 3a. The experimental and fitting error for each value is assumed to be around 10%, but much higher for the ketal resonance due to the low signal to noise ratio. The average $T_{1\rho}^{1H}$ is about 3000 µs ± 900µs. Peak (a) and (a') and (b) and (b'), respectively, were evaluated together due to the fact that the shoulder is not distinguishable from the main resonance for low and very high contact times. Furthermore, both show a similar T_{IS} and $T_{1\rho}^{1H}$.

	ketones (a)	carboxy groups (b)	ketals (c)	peroxides (d)	alcohols (e)	aromatic ipso C	aromatic CH	aliphatic CH, CH_2
T_{IS} / µs	387	1000	1700	365	148	480	101	89
$T_{1\rho}^{1H}$ / µs	3860	2820	3500	3560	3550	3050	2220	1800

Table S4 I_∞ calculated according to equation 2. T_1^{1H} and T_{IS} were taken from Table S3. I_∞ smaller than one shows that the nominal intensity for the individual resonance is not reached for the chosen multiCP conditions. The prediction is made according to the I-S model and does not strictly account for chemical groups with strong heteronuclear dipolar couplings like CH and CH_2 units.

weathering time and multiCP parameters	ketones (a)	carboxy (b)	ketals (c)	peroxide (d)	alcohols (e)	aromatic ipso C	aromatic CH	aliphatic CH, CH_2
0 h, 250µs, 12 blocks τ_{CP}^{tot} = 3 ms	0.965	0.955	0.964	0.962	0.957	0.957	0.926	0.907
1900 h, 250 µs, 8 blocks, τ_{CP}^{tot} = 2 ms	0.965	0.955	0.964	0.962	0.957	0.957	0.926	0.907
2400 h, 150 µs, 13 blocks, τ_{CP}^{tot} = 1.95ms	0.980	0.973	0.979	0.978	0.976	0.975	0.959	0.949
2400 h, 150 µs, 20 blocks, τ_{CP}^{tot} = 3 ms	0.980	0.973	0.979	0.978	0.976	0.975	0.959	0.949
2400 h, 250 µs, 8 blocks, τ_{CP}^{tot} = 2 ms	0.965	0.955	0.964	0.962	0.957	0.957	0.926	0.907
2400 h, 250 µs, 12 blocks, τ_{CP}^{tot} = 3 ms	0.965	0.955	0.964	0.962	0.957	0.957	0.926	0.907
2400 h, 500 µs, 6 blocks, τ_{CP}^{tot} = 3 ms	0.925	0.910	0.929	0.919	0.902	0.910	0.835	0.796
2400 h, 1000 µs, 3 blocks, τ_{CP}^{tot} = 3 ms	0.837	0.818	0.858	0.823	0.787	0.808	0.668	0.604

Table S5 Relative intensities of the PS-^{13}C resonances for the ipso C, the aromatic CH units and the aliphatic CH and CH_2 groups for different weathering times and multiCP conditions before and after correcting with I_∞ given in Table S4. All relative intensities are normalized with respect to the intensity of the ipso carbon. An average intensity ratio of 1: 5.4: 22.0 is observed by combining all multiCP experiments with block lengths of 250 ms and 150 ms.

weathering time and multiCP parameters	ipso C	ipso C / I_∞	aromatic C's	aromatic C's/ I_∞	aliphatic C's	aliphatic C's / I_∞
0 h, 250 µs, 12 blocks τ_{CP}^{tot} = 3 ms	1	1	5.1	5.3	22.4	23.6
1900 h, 250 µs, 8 blocks, τ_{CP}^{tot} = 2 ms	1	1	5.4	5.4	21.8	23.1
2400 h, 150 µs, 20 blocks, τ_{CP}^{tot} = 3 ms	1	1	5.5	5.6	22.6	23.4
2400 h, 250 µs, 12 blocks, τ_{CP}^{tot} = 3 ms	1	1	5.3	5.3	21.1	21.6
2400 h, 250 µs, 8 blocks, τ_{CP}^{tot} = 2 ms	1	1	5.5	5.6	22.3	23.5
2400 h, 500 µs, 6 blocks, τ_{CP}^{tot} = 3 ms	1	1	4.3	4.8	16.1	19.2
2400 h, 1000 µs, 3 blocks, τ_{CP}^{tot} = 3 ms	1	1	4.0	5.1	14.3	20.2

Table S6 Correction factors c_i of the distinct resonances, derived from intensities of the ^{13}C multiCP and CP NMR spectra for the same sample. The multiCP intensities were not corrected with I_∞ before calculating the c_i values.

**functional group**	**PS-^{13}C multiCP 250 ms 8blocks**		**PS-^{13}C multiCP, different parameters, 2400 h**			
	**c_i 2400 h**	**c_i 1900 h**	**1000 µs 3 blocks**	**500 µs 6 blocks**	**250 µs 12 blocks**	**150 µs 20/13 blocks**
ketones	0.73	0.73	0.99	0.93	0.76	0.74
carboxy groups	0.61	0.59	1.04	0.96	0.70	0.64
peroxides	0.73	0.67	1.01	0.95	0.76	0.67
alcohols	0.74	0.65	0.95	0.85	0.69	0.61
ipso C	0.68	0.69	0.94	0.87	0.68	0.67
arom. C´s	0.95	0.91	1.0	0.97	0.92	0.93
aliphatic C´s	1.06	1.07	0.99	1.02	1.06	1.07

Table S7 Correction factors c_i of the distinct resonances, derived from intensities of the ^{13}C multiCP and CP NMR spectra for the same sample. The multiCP intensities were corrected with I_∞ before calculating the c_i values.

functional group	PS-^{13}C multiCP 250 ms 8blocks		PS-^{13}C multiCP, different parameters, 2400 h			
	c_i 2400 h	c_i 1900 h	1000 µs 3 blocks	500 µs 6 blocks	250 µs 12 blocks	150 µs 20/13 blocks
ketones	0.68	0.7	0.76	0.81	0.73	0.72
carboxy groups	0.59	0.56	0.81	0.86	0.67	0.62
peroxides	0.69	0.65	0.78	0.85	0.71	0.65
alcohols	0.71	0.63	0.76	0.76	0.65	0.59
ipso C	0.65	0.66	0.74	0.78	0.7	0.66
arom. C´s	0.94	0.9	0.95	0.94	0.97	0.93
aliphatic C´s	1.07	1.08	1.05	1.04	1.06	1.07

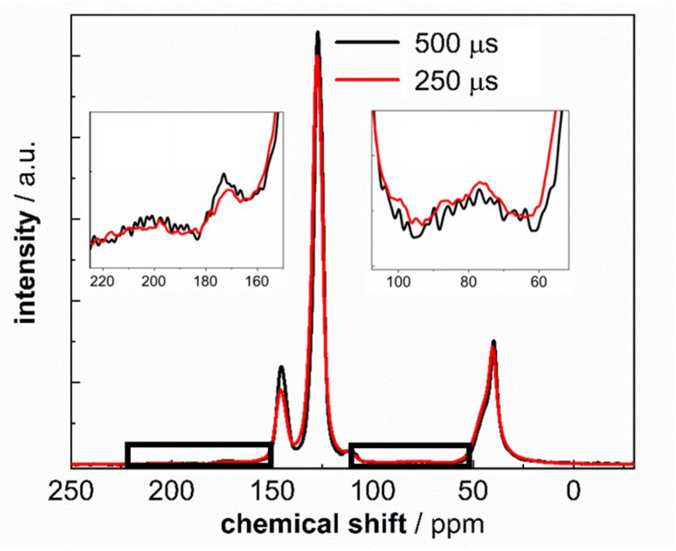

Fig. S10 Comparison of multiCP ^{13}C MAS NMR spectra (red line: $\tau_{multiCP}^{block}$ = 250 µs with 12 blocks and τ_{CP}^{tot} = 3 ms; black line : $\tau_{multiCP}^{block}$ = 500 µs with 6 blocks and τ_{CP}^{tot} = 3 ms) for commercial unadditivated PS weathered for 3200 h. Both spectra are normalised to the same total intensity. The black boxes mark the cut-outs which are shown in the insets.

S2 Calculations of ^{13}C enrichment

One would expect an intensity ratio of around 1:5:20 in the labelled sample. The factor 20 results from one aliphatic CH_2 Carbon and 20% labelled aliphatic CH carbons (corresponding to ca. 19% enrichment). However, the deconvolution of the multiCP spectra for zero weathering (0 h) (see Table S4) gives an average ratio of 1 : 5.1 : 22.4. The relative intensity (normalized to an intensity of 1 per carbon) of the CH, CH_2 resonance at 43 ppm in dependence of the share *c* of the labelled carbons on the total number of carbons at the α-position is given by:

$$1 + (c \cdot 99\% + (1 - c) \cdot 1.1\%) / 1.1\% = 22.4. \quad (3)$$

A share *c* of the CH carbons is labelled and 99% ^{13}C enriched and a share (1-*c*) of the CH carbons occurs in natural abundance (1.1%). The plus 1 stems from the intensity of the CH_2 carbons. This intensity ratio of 22.4 corresponds to a share *c* = 22.8% of labelled carbons and approximately 23.4% ^{13}C enrichment for the aliphatic CH.

S3 Calculation of defect proportions

To calculate the defect proportions, the selective ^{13}C enrichment of 23.4% at the α position of PS needs to be considered. It is assumed that the generated peroxy, carbonyl, carboxy and hydroxy groups are all situated at the labelled C atom due to the stabilisation of benzyl radicals. Thus, their signal intensity will be overestimated by a factor of 21.3, since:

$$(0.228 \cdot 99\% + 0.772 \cdot 1.1\%) / 1.1\% = 21.3 \quad (4)$$

Hence, the defect signal intensities need to be divided by 21.3. For the superimposed aliphatic backbone signals a factor of 11.1 needs to be considered, since:

$$(1.1 + 0.228 \cdot 99\% + 0.772 \cdot 1.1\%) / (1.1\% \cdot 2) = 11.1 \quad (5)$$

Subsequently, the defect proportions were calculated according to Meides et al.[17] with the following equations:

$$p_{ru} = [1 - (8 \cdot I_{ketone}) - (2 \cdot I_{COOH}) - (8 \cdot I_{POOH}) - (8 \cdot I_{OH})] / 8 \quad (6)$$

with:

	p_{ru}	proportion of styrene RUs relative to the proportion of defects
	I_{ketone}	relative intensity of the ketone resonance
	I_{COOH}	relative intensity of the carboxylic acid resonance
	I_{POOH}	relative intensity of the peroxide resonance
	I_{OH}	relative intensity of the alcohol resonance.

The ratio of the different functional groups is $I_{ketone} : I_{COOH} : I_{POOH} : I_{OH} : p_{ru}$. Thus, with the knowledge of p_{ru} the absolute proportions of the carbonyl groups (ap_{ketone}), carboxy groups (ap_{COOH}), peroxy groups (ap_{POOH}) and hydroxy groups (ap_{POH}) can be calculated:

$$ap_{ketone} = I_{ketone} / (p_{ru} + I_{ketone} + I_{COOH} + I_{POOH} + I_{OH}) \quad (7)$$

$$ap_{COOH} = I_{COOH} / (p_{ru} + I_{ketone} + I_{COOH} + I_{POOH} + I_{OH}) \quad (8)$$

$$ap_{POOH} = I_{POOH} / (p_{ru} + I_{ketone} + I_{COOH} + I_{POOH} + I_{OH}) \quad (9)$$

$$ap_{OH} = I_{OH} / (p_{ru} + I_{ketone} + I_{COOH} + I_{POOH} + I_{OH}) \quad (10)$$
